# Comprehensive analysis of cucumber C-repeat/dehydration-responsive element binding factor family genes and their potential roles in cold tolerance of cucumber

**DOI:** 10.1186/s12870-022-03664-z

**Published:** 2022-06-02

**Authors:** Jialin Li, Hongmei Li, Xiaoyan Quan, Qiuli Shan, Wenbo Wang, Ning Yin, Siqi Wang, Zenghui Wang, Wenxing He

**Affiliations:** 1 School of Biological Science and Technology, University of Jinan, Jinan, 250022 China; 2 Shandong Institute of Pomology, Tai’an, Shandong 271000 China

**Keywords:** *CBF* family, Cold tolerance, Cucumber, Expression patterns, Transcriptional regulation

## Abstract

**Background:**

Cold stress is one of the main abiotic stresses limiting cucumber (*Cucumis sativus* L.) growth and production. C-repeat binding factor/Dehydration responsive element-binding 1 protein (CBF/DREB1), containing conserved APETALA2 (AP2) DNA binding domains and two characteristic sequences, are key signaling genes that can be rapidly induced and play vital roles in plant response to low temperature. However, the *CBF* family has not been systematically elucidated in cucumber, and the expression pattern of this family genes under cold stress remains unclear.

**Results:**

In this study, three *CsCBF* family genes were identified in cucumber genome and their protein conserved domain, protein physicochemical properties, gene structure and phylogenetic analysis were further comprehensively analyzed. Subcellular localization showed that all three CsCBFs were localized in the nucleus. *Cis*-element analysis of the promoters indicated that *CsCBFs* might be involved in plant hormone response and abiotic stress response. Expression analysis showed that the three *CsCBFs* could be significantly induced by cold stress, salt and ABA. The overexpression of *CsCBFs* in cucumber seedlings enhanced the tolerance to cold stress, and importantly, the transcript levels of *CsCOR* genes were significantly upregulated in *35S*:*CsCBFs* transgenic plants after cold stress treatment. Biochemical analyses ascertained that CsCBFs directly activated *CsCOR* genes expression by binding to its promoter, thereby enhancing plant resistance to cold stress.

**Conclusion:**

This study provided a foundation for further research on the function of *CsCBF* genes in cold stress resistance and elucidating its mechanism.

**Supplementary Information:**

The online version contains supplementary material available at 10.1186/s12870-022-03664-z.

## Introduction

Low temperature is a major environmental factor affecting the growth and seasonal distribution of field crops and horticultural crops. When plants are exposed to freezing temperatures, their cold tolerance and freezing resistance are increased, that is, this adaptation process is called cold adaptation [1]. Many plants, such as *Arabidopsis* and *oilseed rape*, subjected to low temperature stress, have evolved a complex set of cold-adapted mechanisms involving gene transcriptional regulation and a wide range of physiological, biochemical and metabolic changes [2–4]. In recent years, the molecular mechanism of cold adaptation has been studied extensively and the key regulatory factors of this complex network have been explored. In the *Arabidopsis* genome, 4-20% of the genes are regulated by cold signaling. Many transcription factors including MYB, NAC, MYC, AP2/ERF (APETALA2/ethylene response factor) and bZIP can induce the expression of stress-related genes to protect cells from injury at low temperature [5–7]. Studies have shown that the cold signal regulation pathway dependent on CBF (CRT/DRE binding factor), namely ICE1 (Inducer of *CBF/DREB1* expression 1)-CBF-COR (cold-responsive) pathway, is the most clearly studied cold response pathway in plants [4, 8, 9]. In this pathway, ICE1 is rapidly expressed after low temperature induction and promotes the expression of *CBF* genes and CBFs bind to the CRT/DRE (C-repeat/Dehydration responsive element) *cis*- element on the *COR* gene promoters to activate *COR* gene expression. Thus, plant resistance to low temperature can be improved [10–12].

CBF transcription factors belong to the DREB subfamily of APETALA2/Ethylene-Responsive Factor (AP2/ERF) family, so all CBF proteins have a highly conserved AP2 DNA binding domain, and there are also two conserved characteristic sequences (PKK/RPAGRxKFxETRHP and DSAWR) on both sides of the AP2 domain [13]. The former sequence is directly located in the upstream of AP2 DNA binding domain and may be related to protein transport [13]. *Arabidopsis thaliana* contains six *CBF* genes, namely *AtCBF1* (*DREB1B*), *AtCBF2* (*DREB1C*) and *AtCBF3* (*DREB1A*), *AtCBF4*, *AtDDF1* and *AtDDF2*, among which *AtCBF1*, *AtCBF2* and *AtCBF3* are extensively induced by low temperature and can all improve the cold tolerance of *Arabidopsis thaliana*. These three AtCBF proteins have high homology and are arranged in tandem array as *AtCBF1*-*AtCBF3*-*AtCBF2* on the short arm of chromosome 4 [14–16]. The expression of *AtCBF4* is induced by drought and ABA, but not by low temperature, while overexpression of *AtCBF4* gene can enhance drought tolerance and cold tolerance of *Arabidopsis thaliana* [17]. AtDDF1 and AtDDF2 are related to salt tolerance of *Arabidopsis thaliana* [18].

Currently, *CBF* genes have been isolated from many plant species, such as apple, soybean, rice, tomato, wheat, barley and maize, and have been shown to play important roles in plant cold response [19]. Overexpression of *AtCBFs* in other species can enhance the cold resistance of each species, and the heterogeneous expression of *CBFs* from other plants in *Arabidopsis* can also enhance the cold tolerance of *Arabidopsis* [20–22]. When *TaCBF14* and *TaCBF15* genes from wheat were transferred into barley, the frost tolerance of transgenic plants was higher than that of wild-type barley [23]. Overexpression of *OsDREB1A* could enhance cold resistance of rice [24]. Transgenic potato with *AtCBF1* gene enhanced cold tolerance and induced physiological changes related to adaptation to cold environment [20]. Overexpression of *CsGG3.2* could enhance cold resistance via positively regulated the expression of *CBF* genes in cucumber [25]. Moreover, exogenous melatonin could increase the expression of CBF1 and enhanced the cold tolerance of cucumber seedlings [26]. CBFs are important regulators of plant growth and low temperature response, and the biological function of CBFs in regulating cold tolerance is highly conserved among plants, but also species-specific [27].

Low temperature rapidly activates the expression of *CBF* in plants, which then binds specifically to the DRE/CRT *cis*-element of the *COR* gene promoters to induce its expression [28, 29]. Many *COR* genes have been isolated from plants and are known as *KIN* (cold-inducible), *ERD* (early engend-inducible), *LTI* (low temperature induced) and *RD* (response to dehydration). These genes include *LTI78*, *COR78* (*RD29A*), *COR47*, *COR15A* and *KIN1* [30–33]. In addition to *Arabidopsis*, many *COR* homologues have been cloned from other plants [34]. The expression of *CsCOR1* gene in tea was significantly induced by low temperature and drought, and the heterologous expression of *CsCOR1* in tobacco enhanced its salt tolerance and dehydration tolerance [35]. Under cold stress, two cucumber CBF-inducible *COR* genes, *CsCOR15b* and *CsKIN1*, were higher expressed in *CsGG3.2* overexpression plants [25].

The discovery of CBF transcriptional activators provide a new way to improve plant cold resistance, and lay a theoretical foundation for further discovery of key genes in plant cold resistance mechanism, which has a wide application prospect and important application value in crop and vegetable quality improvement. Cucumber (*Cucumis sativus* L.) is an economically important crop cultivated worldwide [36]. The functions of *CsCBFs* have not been systematically identified in cucumber. In this study, three *CsCBF* genes were identified in cucumber and the comprehensive analyses including the gene structures, conserved domains, phylogenetic analysis and *cis*-elements in promoters were further performed. In addition, the expression patterns of *CsCBFs* under different abiotic stresses were analyzed. Furthermore, overexpressed *CsCBFs* cucumber seedlings increased their cold tolerance by activating *CsCOR* genes expression. These results provide a basis for further research on cold tolerance mechanism of cucumber.

## Results

### Identification and analysis of *CBF* genes in Cucumber

**Fig. 1:**
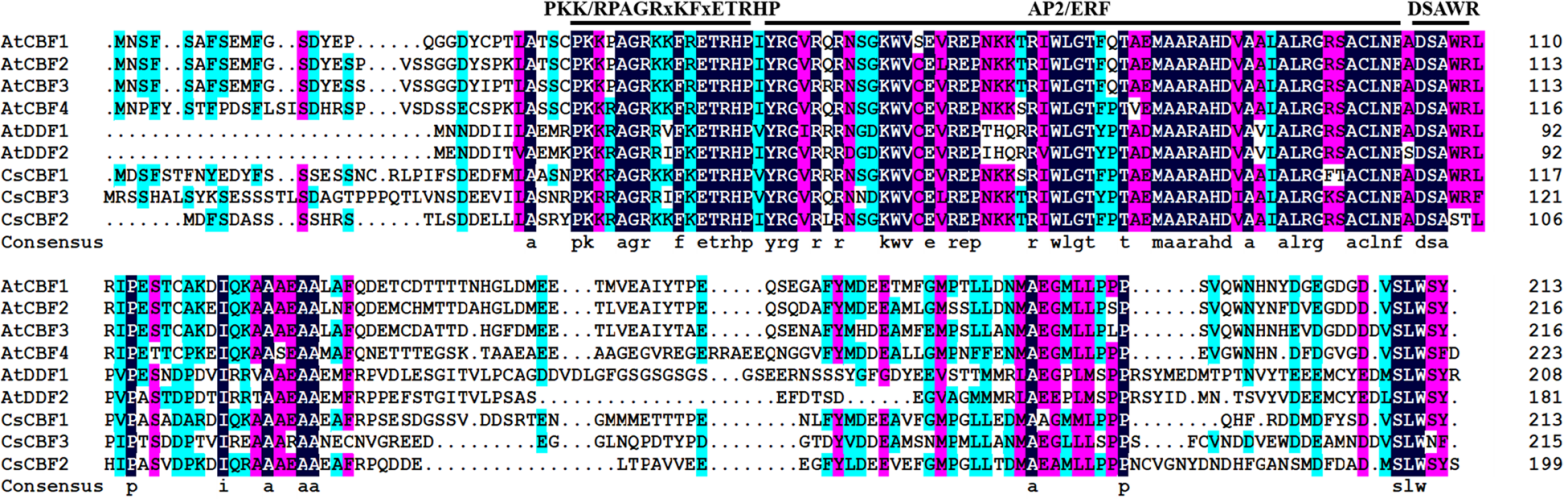
Multiple sequence alignment between CsCBF proteins in cucumber and AtCBF proteins in *Arabidopsis*. The specific conserved APETALA2 (AP2) DNA-binding domains and two characteristic motifs (PKK/RPAGRxKFxETRHP and DSAWR) of CBF proteins were highlighted above the sequence, respectively

To identify *CsCBF* family genes in cucumber genome, the six *Arabidopsis* CBF protein sequences and the AP2/ERF conserved domain and characteristic motif of CBF protein were employed as queries to search against the cucumber genome database using BlastP programme, respectively. Finally, three cucumber *CBF* family genes were identified, and named CsCBF1 to CsCBF3 according to their sequence similarity and phylogenies with individual AtCBF proteins. Multi-sequence alignment (MSA) analysis of the three cucumber CBF protein sequences and six *Arabidopsis* CBF protein sequences showed that the AP2/ERF domain and the characteristic motifs of *CBF* family were highly conserved, and the C-terminal and N-terminal of CsCBF proteins were significantly different (Fig. 1). The information of the *CsCBFs*, including the gene ID, gene name, chromosomal locations, isoelectric points (pI), and amino acid length was shown in Table 1. These three *CsCBF* genes were mapped on chromosome 3, 5 and 5 of cucumber, respectively, and the isoelectric points of CsCBF1, CsCBF2, CsCBF3 protein were 5.10, 5.16 and 4.85, respectively (Table 1). The isoelectric points of all three CsCBF proteins were less than 7, indicating that the three proteins were acidic.

**Table 1:** Information of *CBF* genes in cucumber

**Gene ID**	**Gene name**	**Location**	**Gene length(bp)**	**Amino acid length(aa)**	**pI**
*CsaV3_3G016760*	*CsCBF1*	chr3:12548365 - 12549877	642	213	5.10
*CsaV3_5G005890*	*CsCBF2*	chr5:3899628 - 3900230	603	200	5.16
*CsaV3_5G003250*	*CsCBF3*	chr5:2039646 - 2041467	1822	215	4.85

### Phylogenetic analysis, gene structure and conserved motif analysis of *CBF* genes in cucumber, tomato and *Arabidopsis*

**Fig. 2:**
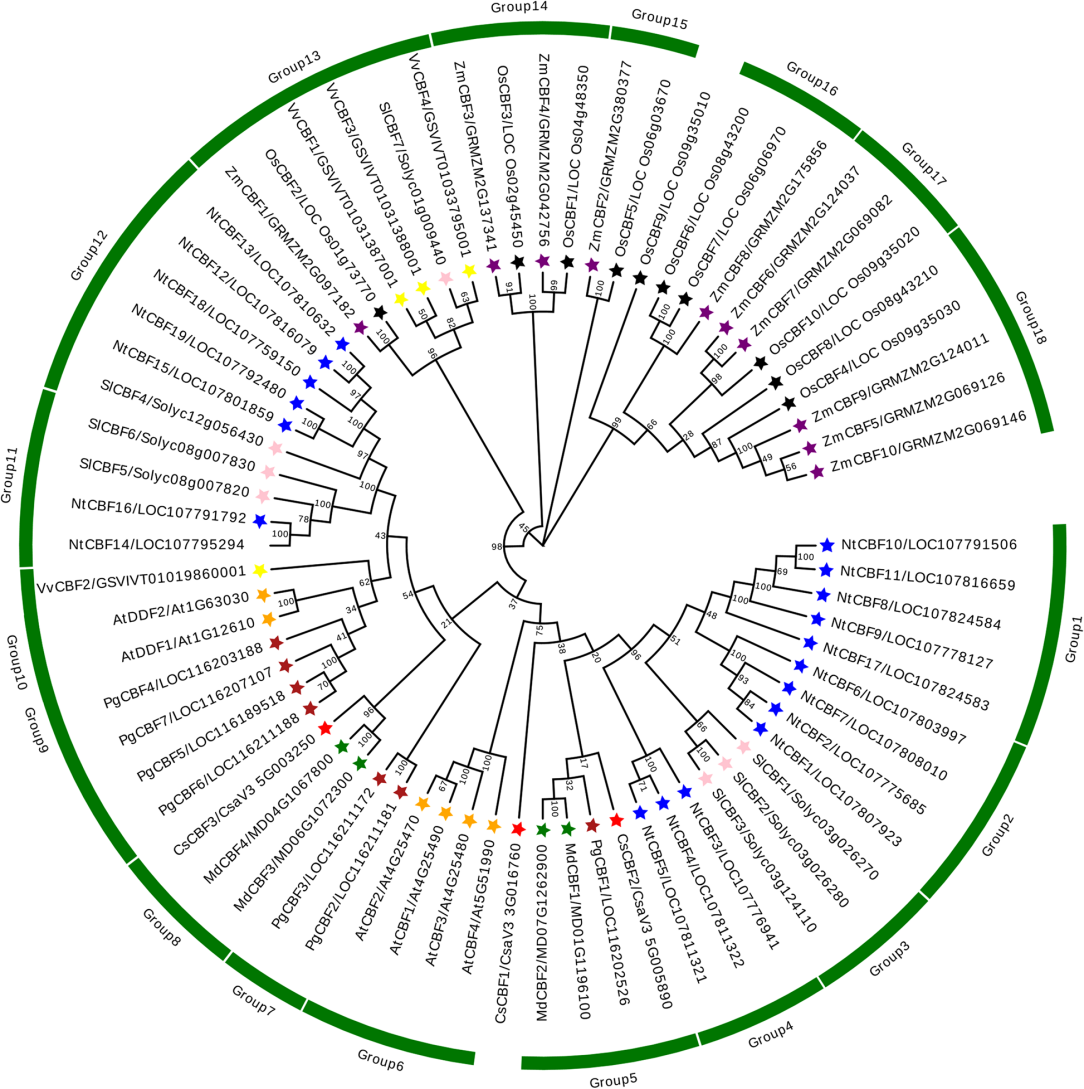
Phylogenetic tree analysis (circle tree) and group classifications of CsCBF1, CsCBF2 and CsCBF3 proteins from cucumber and CBF proteins from other species. The phylogenetic tree was constructed with the full-length amino acid sequences of the 70 CBFs using MEGA 7.0. The analysis included 3 CsCBF proteins (*Cucumis sativus)*, 7 PgCBF proteins (*Punica granatum*), 4 MdCBF proteins (*Malus domestica*), 10 ZmCBF proteins (*Zea mays*), 4 VvCBF proteins (*Vitis vinifera*), 7 SlCBF proteins (*Solanum lycopersicum*), 6 AtCBF proteins (*Arabidopsis thaliana*), 10 OsCBF proteins (*Oryza sativa*) and 19 NtCBF proteins (*Nicotiana tabacum*). The CsCBF proteins were marked with red stars

**Fig. 3:**
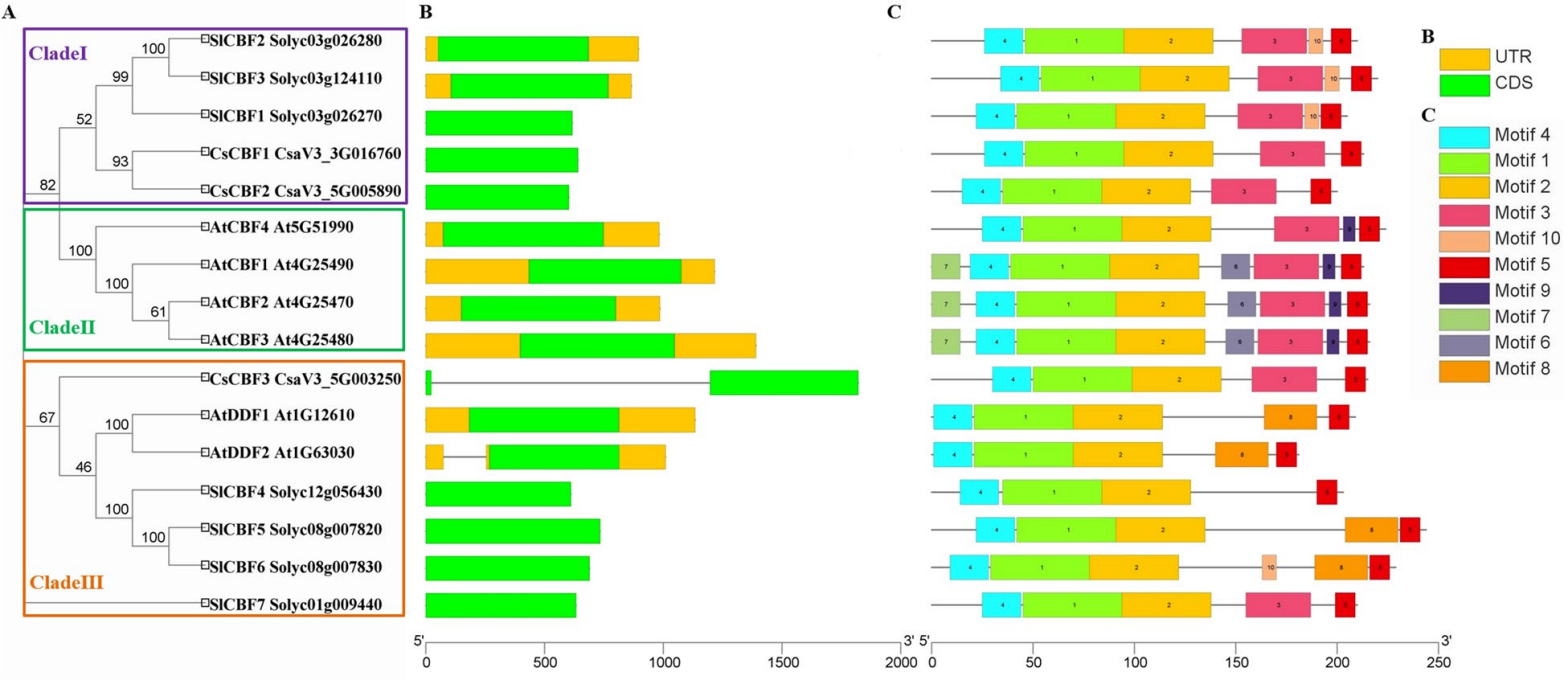
Phylogenetic relationships, gene structure and conserved motif analyses of CBFs from cucumber, tomato and *Arabidopsis*. **A** MEGA 7.0 software was used to construct the phylogenetic tree of 16 CBF proteins and divide into three clades, designated CladeI, CladeII and CladeIII with high bootstrap value. **B** Exon-intron structure of *CBF* genes. **C** The conserved motifs in CsCBFs, SlCBFs and AtCBFs were shown in different colored boxes. The sequences corresponding to each motif were listed in Fig. S1

To analyze orthologous or paralogous relationships of the *CBF* genes from nine plant species, a phylogenetic tree of these genes was constructed from amino acid sequences (Fig. 2). As shown in Fig. 2, the 70 CBF proteins could be roughly divided into 18 groups, of which CsCBF1 and CsCBF2 belonged to group 5 and CsCBF3 belonged to group 8 (Fig. 2; Table S1). To better evaluate the evolutionary relationships of the CsCBF proteins, we further analyzed the phylogenetic tree, gene structures and conserved motifs of six AtCBFs, seven SlCBFs and three CsCBFs (Fig. 3). As shown in Fig. 3A, the resulting tree categorized these CBF proteins into three clades, designated CladeI, CladeII, and CladeIII, and cucumber *CBF* members were classified into Clade I and III. Phylogenetic analysis also revealed that CsCBF1 and CsCBF2 had the highest homology with SlCBF1, SlCBF2 and SlCBF3, and all clustered in Clade I (Fig. 3A). CsCBF3 was classified into CladeIII, which included four SlCBF and two AtCBF proteins, and CladeII only composed of four AtCBF proteins.

The gene structure of *CBFs* from cucumber, *Arabidopsis* and tomato were also analyzed, which was consistent with the results of phylogenetic analysis (Fig. 3B). The number of exons in *AtCBF*, *SlCBF* and *CsCBF* genes was conserved, ranging from one to two exons. We found that the two clades, CladeI and CladeII, had same gene structures, which all contained only one exon and no intron (Fig. 3B). The six proteins, *AtDDF1*, *AtDDF2* and *SlCBF4*-*SlCBF7*, in CladeIII contained one exon, while *CsCBF3* in CladeIII had two exons and one intron (Fig. 3B).

To further analyze the structural diversity and predict the function of the CBF proteins, the motif analysis of them was carried out by MEME (Fig. 3C; Fig. S1). A total of ten distinct motifs, named Motif1-Motif10, were identified (Fig. S1). Motif1, which was representative AP2/ERF domain, and motif 2 and 4, which were the characteristic domains of *CBF* family, were identified in all CBFs. Some of the specific motifs were absent in certain clades. For example, motif 6, 7 and 9 existed only in Clade II, but were absent in all the members of the CladeI and CladeIII. Motif 8 was only identified in CladeIII subfamily, which further corroborated the accuracy of subfamily division. Therefore, the similar motifs distribution of CBFs in these plants might contribute to the prediction of CBF functions.

Collectively, CBF proteins with close evolutionary relationships in the phylogenetic tree generally had similar gene structures and conserved motifs, indicating that evolution of each subfamily in the three different species was relatively conserved.

### *Cis*-elements identification of *CsCBF* gene promoters in Cucumber

**Fig. 4:**
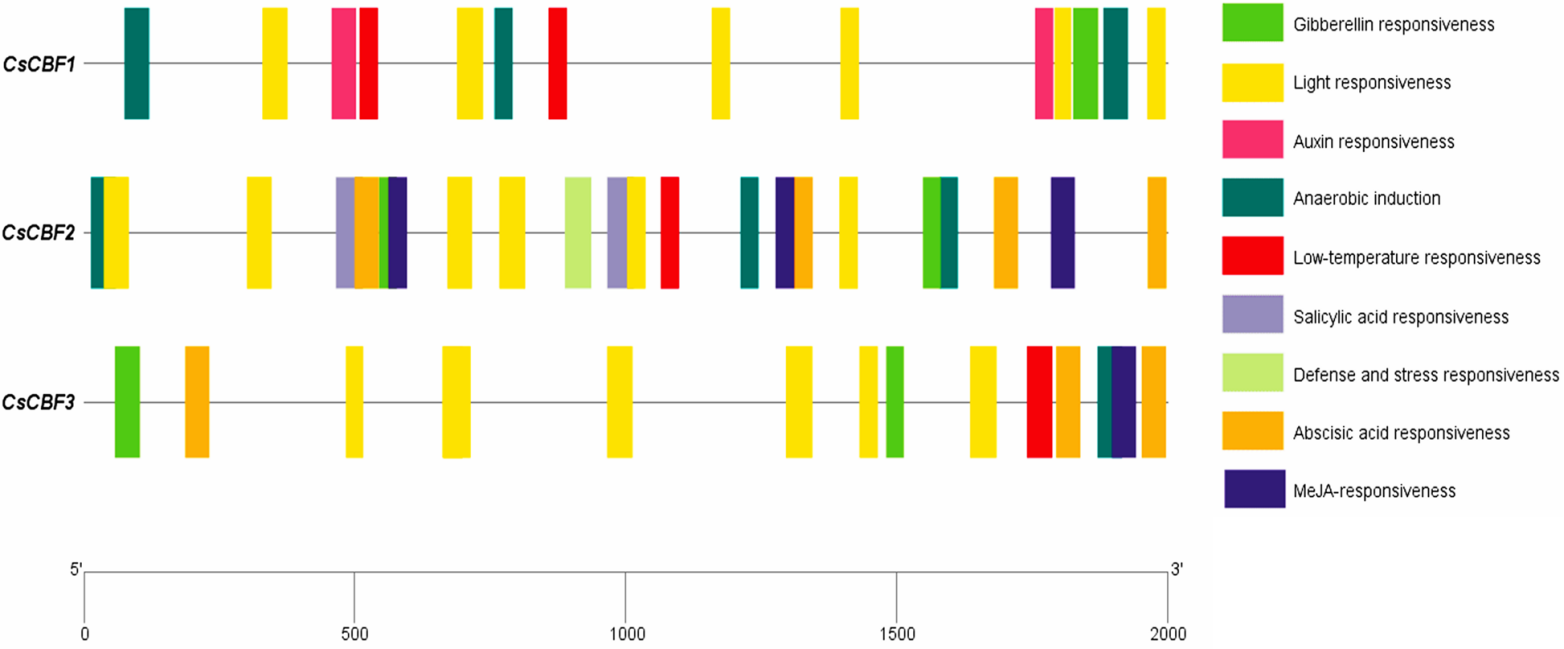
*Cis*-element analysis in the promoter regions of *CsCBF* genes. The 2-kb promoter regions upstream of *CsCBF* genes showed potential *cis*-elements, especially the elements related to stress response (such as light induction, low temperature and anaerobic induction) and plant hormones (such as auxin, abscisic acid and gibberellic acid). Different colored boxes indicated different *cis*-elements

Previous studies have indicated that most of CBF proteins regulate plant growth and tolerance to various abiotic stresses [37]. To investigate the biological function of *CsCBF* genes in cucumber, the potential *cis*-elements were identified on the 2-kb promoter regions of the *CsCBF* genes by PlantCARE (Table S2). As shown in Fig. 4, *cis*-elements responding to hormones such as abscisic acid (ABA), salicylic acid (SA), jasmonate acid (MeJA), auxin and gibberellin (GA) were presented on the promoters of *CsCBF* genes. Moreover, the *CsCBF* promoters also contained *cis*-elements in response to abiotic stresses, such as low temperature, defense and stress. In addition, the three *CsCBF* gene promoters all had light response signal elements (Fig. 4), suggesting that *CsCBF* genes might also be involved in the regulation of cucumber growth and development by light signal. In conclusion, the three *CsCBF* genes in cucumber may be involved in the response to multiple plant hormones and abiotic stresses.

### Expression patterns of *CsCBFs* under different abiotic stresses

**Fig. 5:**
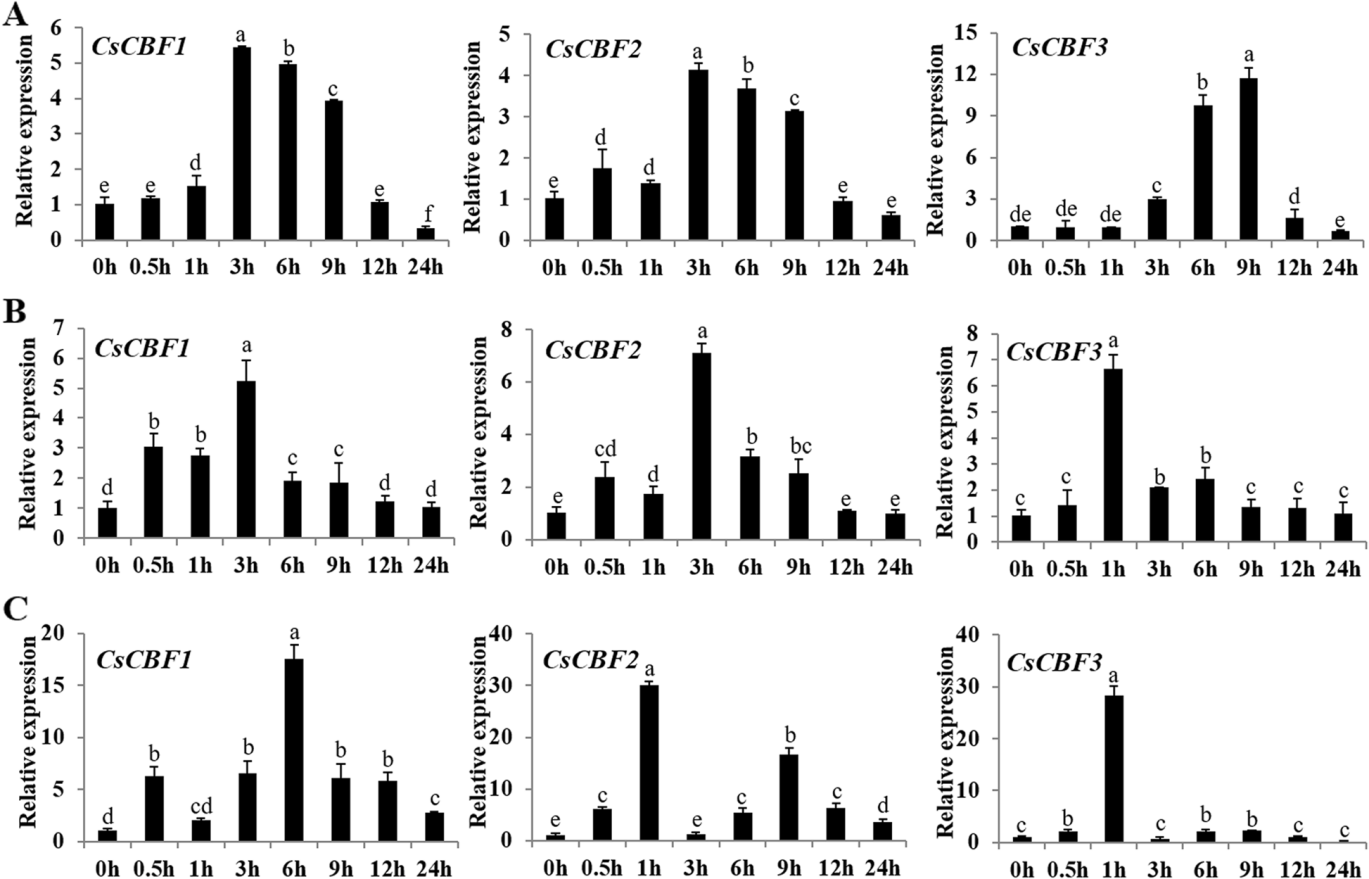
Expression patterns of *CsCBF1*, *CsCBF2* and *CsCBF3* in cucumber under different abiotic stresses. **A** low temperature (4 °C) treatment. **B** 100 mM NaCl treatment. **C** 100 μΜ ABA treatment. The extracted RNA samples were collected at 0.5h, 1h, 3h, 6h, 9h, 12h and 24h after the corresponding treatment, respectively. The cucumber *β-actin* gene was used as an internal control, and three biological replicates were used for gene expression analyses. Error bars were the standard errors (SE). Different lowercase letters represented significant differences (*P* < 0.05)

To investigate whether *CsCBF* genes respond to abiotic stress in cucumber, qRT-PCR was used to detect the expression levels of *CsCBF* genes under different stress conditions including low temperature (4°C), salt (100 mM NaCl) and ABA (100 μΜ ABA) (Fig. 5). Under low temperature treatment, the expression patterns of *CsCBF1* and *CsCBF2* showed a similar trend, which was firstly rapidly increased, and reached maximum values at 3 h, decreasing thereafter (Fig. 5A). However, the expression levels of *CsCBF3* reached the maximum at 9 h, suggesting that *CsCBF3* was less sensitive to low temperature than *CsCBF1* and *CsCBF2*. Similar to the expression pattern after low temperature treatment, the expression levels of *CsCBF1* and *CsCBF2* achieved the maximum after 3 h of salt stress, while the expression level of *CsCBF3* was highest after just 1 h of NaCl treatment, then rapidly decreased to the initial level and remained at a low level all the time (Fig. 5B). Different from the above treatments, *CsCBF* genes responded to ABA more rapidly, and all significantly increased at 0.5 h. The expression levels of *CsCBF2* and *CsCBF3* increased rapidly at 0.5h and reached the maximum at 1 h, then dropped to the initial level at 3h and increased again at 6h, decreasing thereafter (Fig. 5C). Unlike *CsCBF2* and *CsCBF3*, the expression level of *CsCBF1* reached its highest value at 6h of ABA treatment (Fig. 5C). These results suggested that *CsCBFs* were involved in response to low temperature, salt and ABA.

### Subcellular Localization of CsCBFs

**Fig. 6:**
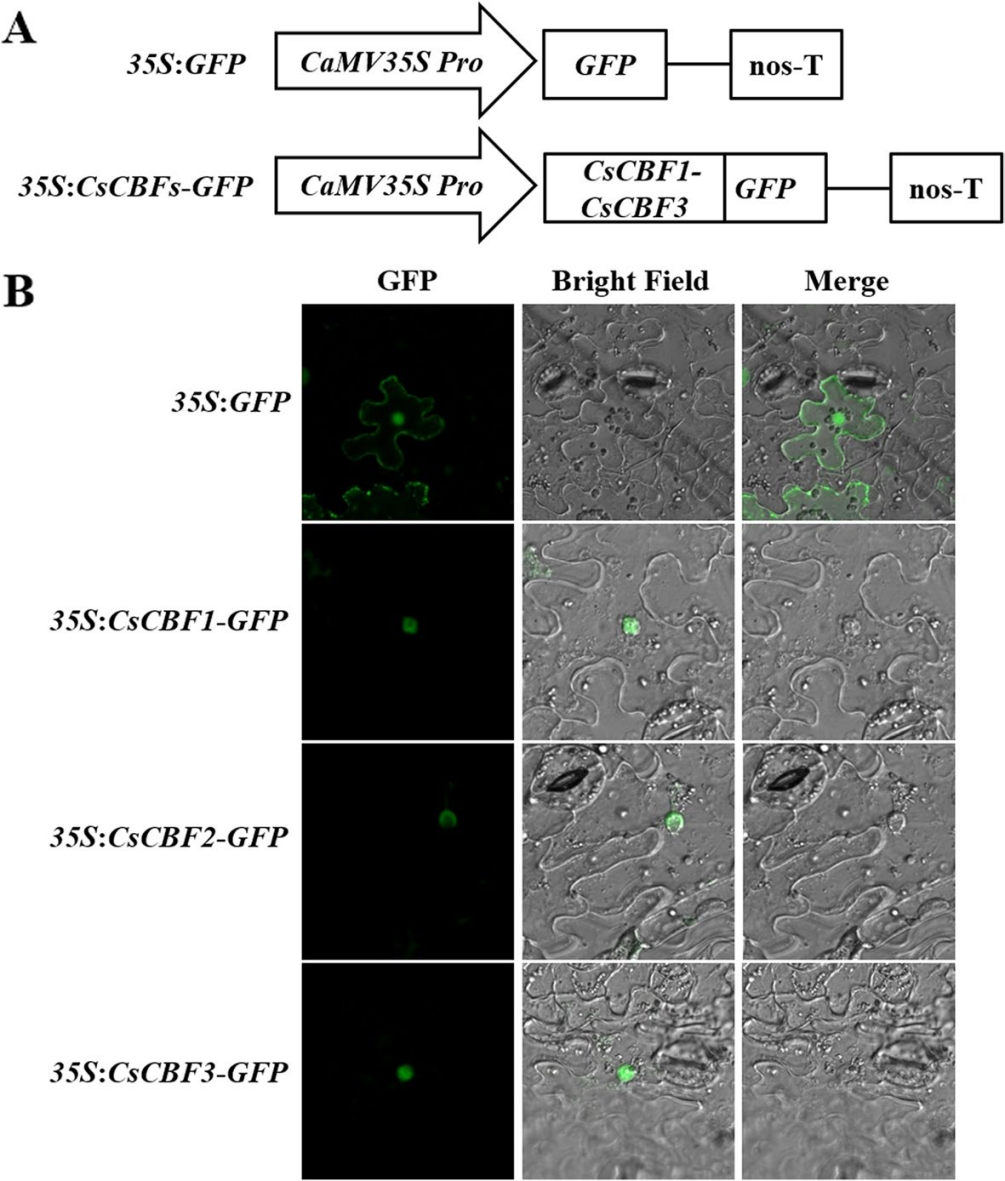
Subcellular localization analysis of CsCBF1, CsCBF2 and CsCBF3 proteins in tobacco leaf cells. **A** Schematic diagram of the control (*35S*:*GFP*) and *35S*:*CsCBFs*-*GFP*. **B** Transient expression of *35S*:*GFP* and *35S*:*CsCBFs*-*GFP* in tobacco leaf. After 48 h of transformation, green fluorescence signal was observed under confocal microscope

PredictProtein software was used to predict the subcellular localization of CsCBF1, CsCBF2 and CsCBF3 proteins [38], and the prediction result showed that CsCBF1, CsCBF2 and CsCBF3 proteins were all in the nucleus (Fig. S2). To verify the predicted results of CsCBF proteins, the fusion protein vectors *35S*:*CsCBF1*-GFP, *35S*:*CsCBF2*-GFP and *35S*:*CsCBF3*-GFP were constructed, respectively (Fig. 6A). The GFP emited a green fluorescent signal under a laser-scanning confocal microscopy to determine where the gene is expressed. Microscopically, epidermal cells from tobacco leaves expressing the different CsCBF fusion proteins all only showed a fluorescence signal in the nucleus. As a control, the *35S*:*GFP* fluorescence was observed throughout the whole cell (Fig. 6B). These data indicated that CsCBF proteins were all nuclear localization proteins, which were consistent with the previous prediction. The three CsCBF transcription factors may play roles in transcriptional regulation.

### Overexpression of *CsCBFs* enhanced the tolerance of transgenic cucumber seedlings to cold stress

**Fig. 7:**
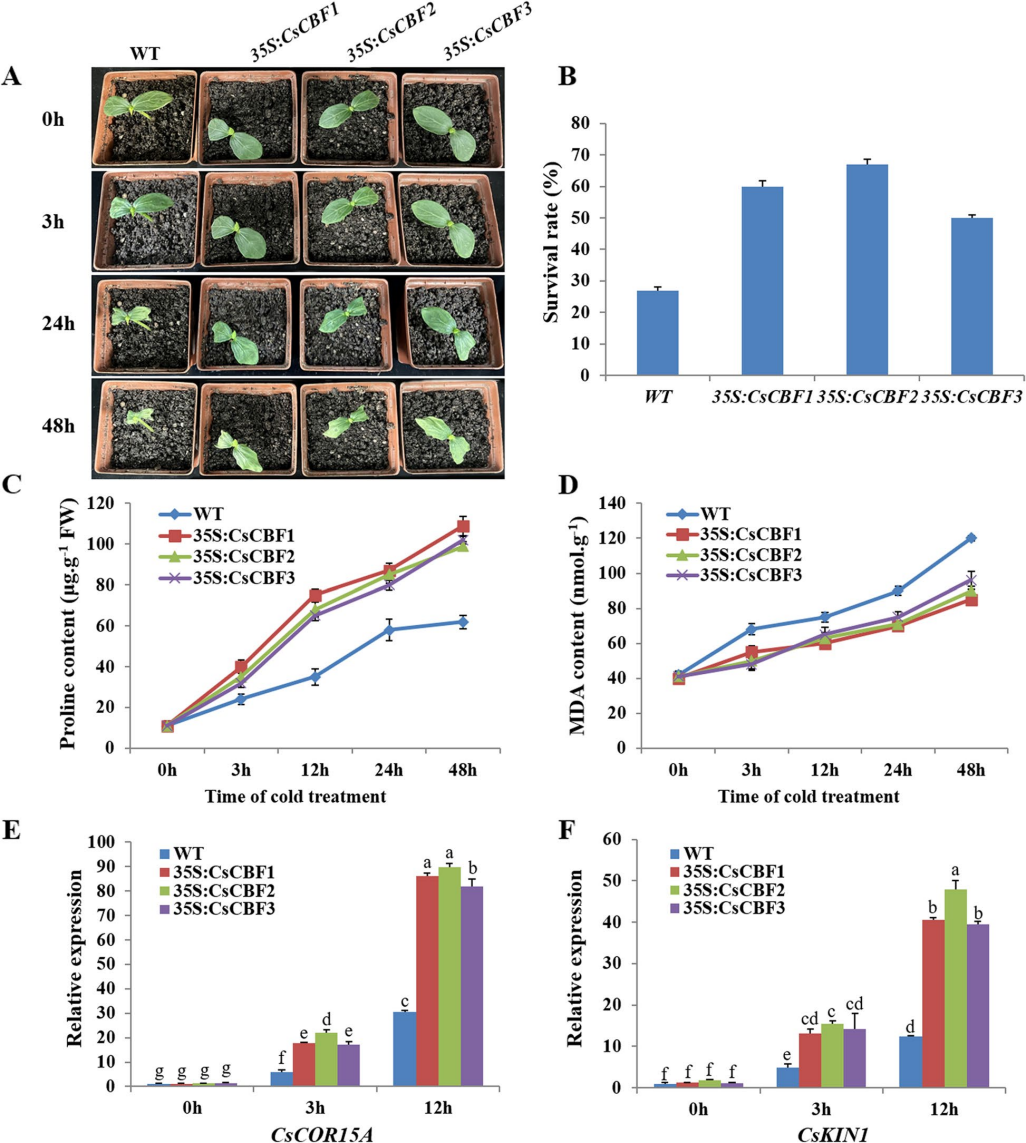
Overexpression of *CsCBFs* improved cold tolerance of transgenic cucumber seedlings. **A** The phenotypic analysis of WT (*35S* empty vector) and *35S*:*CsCBFs* overexpressing cucumber seedlings treated under 0°C for different time periods. **B** Survival rates of WT and *35S*:*CsCBFs* transgenic plants after 48 h cold treatment. The *35S*:*CsCBFs* transgenic cucumber seedlings all showed higher proline contents (**C**) and lower MDA contents (**D**) than WT under 0°C for different time periods. **E**, **F** The expression changes of *CsCOR15A* and *CsKIN1* genes in *35S*:*CsCBFs* transgenic plants and WT under 0°C cold treatment, respectively. Three biological replicates were performed for gene expression analyses. The bars showed the SE. Different lowercase letters represented significant differences (*P* < 0.05)

The expression levels of *CsCBFs* were significantly induced by low temperature (Fig. 5). In order to further investigate the response of *CsCBF1*, *CsCBF2* and *CsCBF3* to cold stress and their biological functions, the *agrobacterium*-mediated transient transformation experiments were conducted in cucumber cotyledons to clarify *CsCBFs* tolerance to cold stress. Cucumber seedlings overexpressing *CsCBFs* with GFP fluorescence signal in cotyledons were selected for subsequent experiments (Fig. S3A). The qRT-PCR analysis showed that the expression of *CsCBF* genes in their transgenic cucumbers were significantly higher than those in WT plants (overexpressing *35S* empty vector) (Fig. S3B), and different stress treatments all promoted the expression of *CsCBF* genes in transgenic cucumber (Fig. S3C-E). To test whether *CsCBF*s can enhance cold resistance of cucumber cotyledons, transgenic cucumber seedlings and WT were treated at 0°C, respectively. Before treatment, the *35S*:*CsCBFs* transgenic plants and WT all grew well, while after 3 h of cold treatment, slight wilting appeared in cotyledons of WT compared with transgenic seedling overexpressing *CsCBF1*, *CsCBF2* and *CsCBF3*, and serious wilting in WT showed more obvious difference from all transgenic seedlings after 24 h (Fig. 7A). After 48 h, the survival rate of WT was only 27%, while the survival rates of the *35S*:*CsCBF1*, *35S*:*CsCBF2* and *35S*:*CsCBF3* transgenic plants were 60%, 67% and 56% (Fig. 7A-B).

The contents of proline and MDA are important physiological indexes to measure cold resistance of plant [39]. Compared with WT plants, the *35S*:*CsCBFs* transgenic plants all showed a significant increase in proline and great decrease in MDA content (Fig. 7C-D). In addition, to verify that overexpression of *CsCBFs* enhanced cold tolerance of cucumber seedlings by regulating *COR* genes, the expression levels of two *CsCOR* genes (*CsCOR15A* and *CsKIN1*) in *CsCBFs* overexpressed plants and WT were detected under cold treatment, respectively. As shown in Fig. 7E-F, the transcriptional levels of the two genes in transgenic plants were significantly higher than those in WT plants after cold treatment. Based on survival rate, physiological indexes and *CsCOR* genes expression, *CsCBF1* and *CsCBF2* played stronger roles in cold stress response than *CsCBF3* (Fig. 7). Taken all together, these results suggested that overexpression of *CsCBF* genes in cucumber could significantly enhance cold tolerance of cucumber.

### CsCBFs directly activate *CsCORs* expression by binding to their promoters

**Fig. 8:**
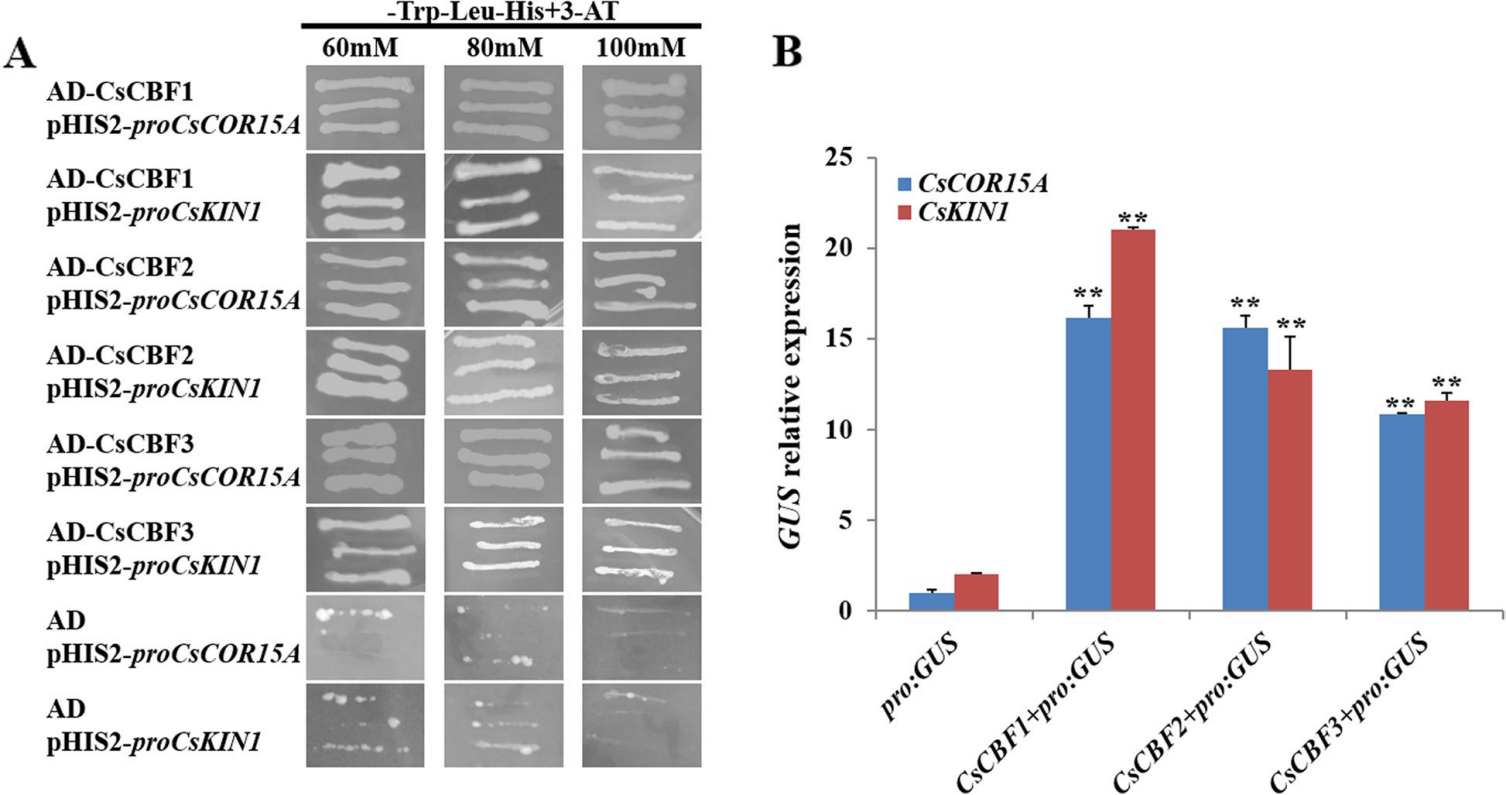
CsCBFs directly bound to the promoters of *CsCOR15A* and *CsKIN1*. **A** CsCBF1, CsCBF2 and CsCBF3 proteins all could bind to the promoters of *CsCOR15A* and *CsKIN1* in yeast-one-hybrid assay. **B** *GUS* transient expression assays showing that CsCBF1, CsCBF2 and CsCBF3 proteins all activated the expression of *CsCOR15A* and *CsKIN1*, respectively. Three biological replicates were performed for gene expression analyses. Error bars were the standard errors (SE). Statistical significance: **P < 0.01

Overexpression of *CsCBFs* greatly induced the expression of *CsCORs* (Fig. 7E-F), and previous reports have shown that CBFs can directly bind to the promoter regions of *COR* genes to activate their expression [12]. To verify whether CsCBFs can bind to the promoters of *CsCOR*s, the 2-kb promoter fragments of *CsCOR15A* and *CsKIN1* were selected and inserted into the pHIS2 plasmid, respectively. The CDSs of *CsCBFs* were separately cloned into the pGADT7 vector. The yeast one-hybrid (Y1H) assays were carried out and the results showed that CsCBF1, CsCBF2, and CsCBF3 proteins all could specifically directly bind to the promoters of *CsCOR15A* and *CsKIN1*, but not empty pHIS2 vector (Fig. 8A). The transient *GUS* activity assays were carried out in tobacco leaves to verify the above results. The above DNA fragments were separately inserted into pCAMBIA1300-*GUS* vector containing *GUS* reporter gene, and the CDSs of *CsCBFs* were cloned into the pCAMBIA1300 plasmid to obtain *35S*:*CsCBFs* recombinant plasmids. The results showed that all three CsCBFs could activate the expression of *CsCOR15A* and *CsKIN1* in vivo (Fig. 8B). These data revealed that CsCBFs could directly activate *CsCORs* expression by binding to their promoters.

## Discussion

Cucumber (*Cucumis sativus* L.) is an economically important crop cultivated worldwide and one of the main vegetables grown in the facility [36]. Low temperature is a major environmental factor affecting the growth and seasonal distribution of cucumber. The transcription factor CBFs (C-repeat Binding Factor) are key "molecular switch" for plant to sense low temperature signals and regulate their adaptive responses. These genes can activate the expression of several downstream cold-tolerance related functional genes to improve plant resistance to low temperature [40]. Therefore, bioinformatics analysis of *CBF* gene family in cucumber was conducted to provide theoretical basis for analyzing the mechanism of *CBFs* regulating cucumber cold stress response.

Studying the differences in gene structure and conserved motifs is an important reference for analyzing the evolutionary relationships of gene families [33]. In our study, multi-sequence alignment analysis of CsCBFs showed that the three CsCBF proteins all contained conserved AP2 domains and their flanks (Fig. 1). By analyzing the gene structures of *CsCBFs*, it was found that the three *CsCBF* genes in cucumber did not contain UTR (Fig. 3). Phylogenetic tree analysis showed that 70 CBF proteins from cucumber and other species were divided into 18 subgroups. Among them, the three CsCBF proteins are closely related to CBF proteins in apple and pomegranate (Fig. 2). Previous studies have shown that *CBF* genes play important roles in plant growth and development. Overexpression of *AtCBFs* results in plant growth retardation and delayed flowering [16, 41]. Here, we showed that light response signal elements were found on the promoters of the three *CsCBF* genes (Fig. 4), suggesting that *CsCBFs* may be involved in the regulation of light signal in the growth and development of cucumber.

Studies have shown that light is also an important environment, and photochrome-interacting factors (PIFs) play a key role in regulating plant development [42]. Part PIFs can bind to G-box and E-box *cis*-elements in *AtCBF* promoters to regulate their transcription, and the *PIF3/4/7* are negatively involved in the low temperature response pathways of plants and can negatively regulate *CBF* expression [43]. In our study, *cis*-elements that respond to abiotic stresses and hormones such as low temperature, defense and stress, abscisic acid, salicylic acid, jasmonate acid, auxin and gibberellin were simultaneously screened on promoters of *CsCBF* genes (Fig. 4). The expression analysis showed that *CsCBFs* were indeed regulated by low temperature (Fig. 5A), which will help further elucidate the molecular regulation mechanism of cold signaling.

The functions of *CBF* genes in apple, soybean, rice, tomato, wheat, barley and maize have been widely reported [19, 44–46]. Overexpression of *DREB* can enhance plant resistance to stress, and *35S*:*PpDBF1* transgenic tobacco has higher salt tolerance, drought tolerance and cold tolerance [47]. The heterologous expression of *ZmCBF3* and *CsCBF3* (tea) in *Arabidopsis* can significantly enhance the frost resistance of *Arabidopsis thaliana* [48, 49]. Overexpression of *CsCBFs* in the cotyledons of cucumber seedlings could markedly enhance its cold resistance and the changes of Pro and MDA in *35S*:*CsCBFs* transgenic cucumber seedlings indicated that *CsCBFs* could enhance cold tolerance of cucumber (Fig. 7). Low temperature induces the expression of *CBFs*, thereby activating the expression of downstream target genes. Heterologous expression of *VvCBF1* in *Arabidopsis* can enhance the expression of *AtCOR15A*, *AtRD29B* and *AtRD29A* [50]. The expression of *AtCOR15A*, *AtCOR47*, *AtKIN1* and *AtRD29A* in *35S*:*SmCBFs* transgenic *Arabidopsis thaliana* were up-regulated, thus enhancing its cold resistance [51]. In this study, the transcript level of *CsCOR* genes was significantly upregulated in *35S*:*CsCBFs* transgenic plants after cold stress treatment (Fig. 7). Y1H and *GUS* experiments demonstrated that CsCBFs, as transcription factors, could directly bind to promoters of *COR* genes and activate their expression (Fig. 8). These results provided a foundation for further research on the function of *CsCBFs* gene in cold stress resistance and elucidating its mechanism.

## Conclusions

In this study, we comprehensively analyzed the cucumber *CBF* family genes. The expression patterns of *CsCBF* genes under different stress treatments were also investigated, and the roles of *CsCBFs* in cucumber cold tolerance were analyzed in detail by transient transgenic method. This study provided a foundation for further research on the function of *CsCBF* genes in cold stress resistance and elucidating its mechanism.

## Methods

### Genome-wide identification of *CsCBFs* in cucumber

To identify the *CsCBF* genes from cucumber genome database (http://cucurbitgenomics.org/organism/20), six *Arabidopsis* AtCBF proteins were used as query sequences and Blastp was used to search for the predicted proteins. All candidate genes were further confirmed by the existence of conserved characteristic sequences (PKK/RPAGRxKFxETRHP and DSAWR) and AP2 (PF00847.20) domains using the Pfam (available online: http://pfam.janelia.org) and Simple Modular Architecture Research Tool (SMART) datebase (http://smart.embl-heidelberg.de).

### Physicochemical properties of CsCBF proteins

ExPASy software (http://web.expasy.org/protparam/) was used to analyze protein sequences of CsCBFs to predict amino acid length and isoelectric point (pI). The location of *CsCBF* genes on cucumber chromosome was determined according to the physical location information in cucumber genome database.

### Phylogenetic analysis, gene structure and conserved motif analysis

Multiple sequence alignments of these proteins were performed using ClustalW with default parameters. An un-rooted phylogenetic tree was constructed with the full-length amino acid sequences of the 70 CBFs using MEGA 7.0, and the neighbour-joining (NJ) method was used with the following parameters: Poisson correction, pairwise deletion, and bootstrap (1000 replicates; random seed) [52]. The corresponding DNA and cDNA sequences of each predicted gene were downloaded from genomes, and the gene structures were analyzed as described by [53]. The conserved motifs in CsCBFs were identified using Multiple Expectation Maximization for Motif Elicitation (MEME) online program (http://meme-suite.org/index.html) [53].

### Identification of *cis*-elements on *CsCBFs* promoter in cucumber

The entire cucumber genome data were downloaded from the cucumber genome database (Chinese Long 9930: http://cucurbitgenomics.org/), and the 2 kb sequences upstream of the transcription start site of of *CsCBFs* were extracted by TBtools. The *cis*-elements on the promoter regions of *CsCBF* genes were analysed by PlantCARE website (http://bioinformatics.psb.ugent.be/webtools/plantcare/html/) [52].

### Plasmid construction and transient transformation of cucumber cotyledons

To generate *35S*:*CsCBFs*-*GFP*, the full-length coding sequence of *CsCBF1*, *CsCBF2* and *CsCBF3* were amplified and cloned into pCAMBIA1300 vector with a GFP tag, respectively. The recombinant plasmids were transformed into *Agrobacterium tumefaciens* LBA4404, and then transferred into 8-d-old cucumber cotyledons for subsequent cold tolerance tests [54]. The primers used are listed in Table S3.

### Subcellular localization of CsCBFs

To determine the subcellular localization of CsCBFs, the empty GFP vector and the recombinant plasmid of *35S*:*CsCBF1*-*GFP*, *35S*:*CsCBF2*-*GFP* and *35S*:*CsCBF3*-*GFP* were injected into tobacco leaf epidermal cells, respectively. The injected tobaccos were grown under normal conditions for about 48 hours. The fluorescent signal was observed by a fluorescence microscope.

### Expression pattern of *CsCBF* genes under different abiotic stresses

The cucumber inbred line Xintaimici was used for transient genetic transformation and stress treatments, and all plants were cultured in a light incubator under 28 °C with 16 h light /20 °C with 8 h dark cycle conditions. The two-week-old cucumber seedlings were placed in a 4 °C incubator for low temperature treatment until the leaves were collected, and the leaves of two-week-old cucumber seedlings with consistent growth were sprayed with 100 μM ABA and 100 mM NaCl, respectively. The leaves were selected at 0, 0.5, 1, 3, 6, 9, 12 and 24h for subsequent quantitative analysis. The mixed leaves of five plants were one replicate, and each sample contained three biological replicates. Each treatment was repeated at least three times.

### Yeast one-hybrid assays

The 2-kb promoter fragments of *CsCOR15A* and *CsKIN1* were selected and inserted into the pHIS2 plasmid, respectively. The coding sequences (CDSs) of *CsCBFs* were separately cloned into the pGADT7 vector to obtain the constructs AD-CsCBFs. The optimal 3-AT concentration which could inhibit the growth of background histidine of pHIS2 vector was screened. Then the recombinant pHIS2 vector and AD-CBFs were co-transferred to yeast strain Y187 and grown on medium SD/-Trp-Leu-His with optimal 3-AT concentration. Empty vector pGADT7 was used as the control. Y1H was conducted according to [55]. The primers used are listed in Table S3.

### Transient *GUS* activity assays

The ORFs of *CsCBFs* were separately inserted into the pCAMBIA1300 vector. The 2kb promoter fragments of *CsCOR15A* and *CsKIN1* were separately inserted into pCAMBIA1300-*GUS* plasmids to obtain *proCsCOR15A*:*GUS* and *proCsKIN1*:*GUS* recombinant plasmids. The tobacco leaves were used to conduct *GUS* activity assays. The different combinations were injected into 5-week-old tobacco leaves by agrobacterium-mediated method. Under normal conditions, the injected tobacco grew 2-3 days for subsequent experiments. The transient activity assays were measured as described previously [54].

## Supplementary Information

**Additional file 1:** **Figure S1.** The logos represented the 10 conserved motifs of CBF proteins, which were derived from MEME Suite. **Figure S2.** The subcellular localization of CsCBF1, CsCBF2 and CsCBF3 proteins were predicted using PredictProtein software, which were all located in the nucleus. **Figure S3.** Expression levels of *CsCBF1*, *CsCBF2 *and *CsCBF3 *in *35S*:*CsCBFs *transgenic plants.

**Additional file 2: Table S1.**

**Additional file 3: Table S2.**

**Additional file 4: Table S3.**

## Data Availability

The data that support the results are included within the article and its additional files. Other relevant materials are available from the corresponding authors on reasonable request.
