# Supplementary material for: Comprehensive analysis of cucumber C-repeat/dehydration-responsive element binding factor family genes and their potential roles in cold tolerance of cucumber
Source: BMC Plant Biol. 2022 Jun 2;22:270. doi: 10.1186/s12870-022-03664-z (PMC9161515; doi:10.1186/s12870-022-03664-z)
Supplement: Supplementary file 1 — Additional file 1: Figure S1. The logos represented the 10 conserved motifs of CBF proteins, which were derived from MEME Suite. Figure S2. The subcellular localization of CsCBF1, CsCBF2 and CsCBF3 proteins were predicted using PredictProtein software, which were all located in the nucleus. Figure S3. Expression levels of CsCBF1, CsCBF2 and CsCBF3 in 35S:CsCBFs transgenic plants. [file 12870_2022_3664_MOESM1_ESM.pdf]

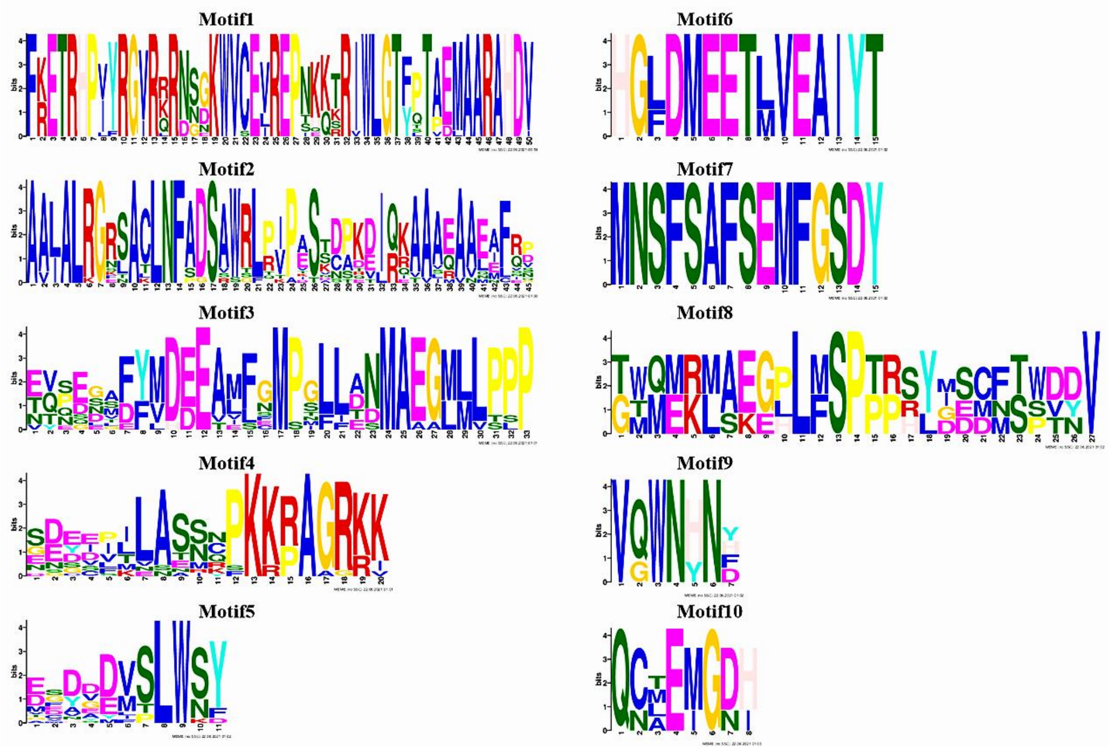

Fig. S1. The logos represented the 10 conserved motifs of CBF proteins, which were derived from MEME Suite.

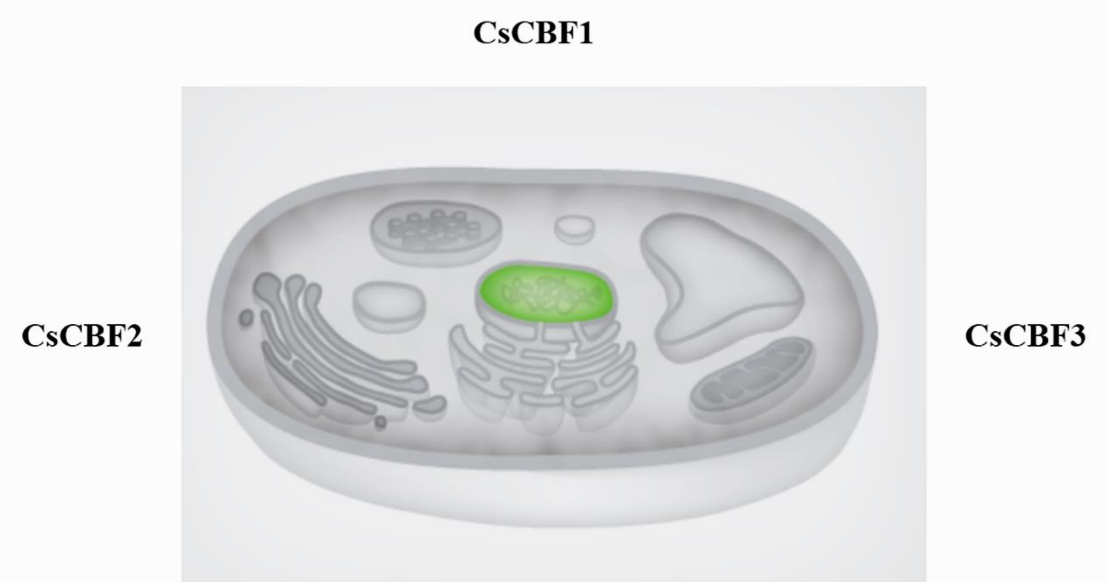

Fig. S2. The subcellular localization of CsCBF1, CsCBF2 and CsCBF3 proteins were predicted using PredictProtein software, which were all located in the nucleus.

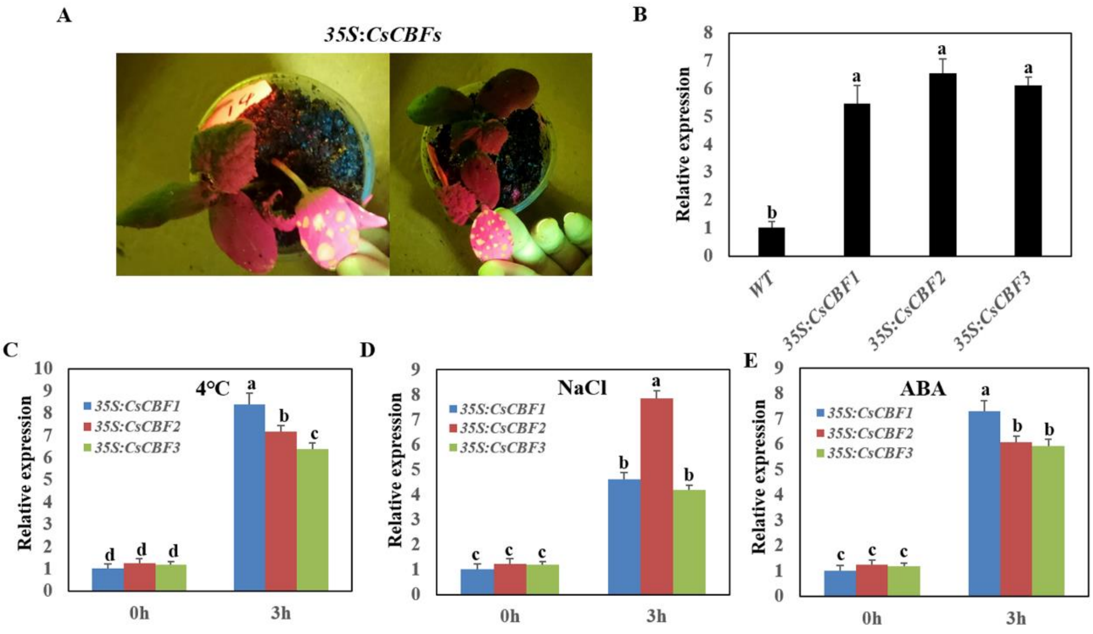

Fig. S3. Expression levels of *CsCBF1*, *CsCBF2* and *CsCBF3* in *35S*:*CsCBFs* transgenic plants. (A) The cotyledons of transgenic plants showed GFP fluorescence under the GFP channel of portable fluorescent protein excitation light source (LUYOR-3415RG). (B) Relative expression levels of *CsCBF1*, *CsCBF2* and *CsCBF3* in *35S*:*CsCBFs* transgenic plants and WT (overexpressing *35S* empty vector). (C-E) The expression changes of *CsCBF1*, *CsCBF2* and *CsCBF3* genes in *35S*:*CsCBFs* transgenic plants under 4℃, NaCl, ABA treatment for 3h, respectively. The cucumber *β-actin* gene was used as an internal control, and three biological replicates were used for gene expression analyses. Error bars were the standard errors (SE). Different lowercase letters represented significant differences ($P < 0.05$).
